# BIGrat: a repeat resolver for pyrosequencing-based re-sequencing with Newbler

**DOI:** 10.1186/1756-0500-5-567

**Published:** 2012-10-15

**Authors:** Tongwu Zhang, Yingfeng Luo, Yaping Chen, Xiaonuan Li, Jun Yu

**Affiliations:** 1 CAS Key Laboratory of Genome Sciences and Information, Beijing Institute of Genomics, Chinese Academy of Sciences, Beijing 100029, China; 2 James D. Watson Institute of Genome Sciences, College of life Science, Zhejiang University, Hangzhou 310058, China

## Abstract

**Background:**

As more and more reference genome sequences are assembled, it becomes practical to assemble individual genomes from large amount of raw read data based on a reference sequence. However, most available assembly tools are designed for *de-novo* genome assembly. There is one commercial tool box (Newbler) developed for re-sequencing projects based on the Roche 454 sequencing platform. However, the genome with large repeat regions cannot be well assembled in Newbler.

**Findings:**

We developed a new sequence assembly tool (BIGrat, Beijing Institute of Genomics Re-Assembly Tool) for pyrosequencing-based re-sequencing projects, such as data generated from Roche 454 and IonTorrent platforms. BIGrat improves the output of Newbler when evaluated on genome assemblies including chloroplast, mitochondrial, bacterial, and plant nuclear genomes.

**Conclusion:**

We presented a novel sequence assembly tool BIGrat for pyrosequencing-based re-sequencing projects, which can easily be integrated into Newbler pipelines for next-generation sequencing assembly and analysis.

## Introduction

Together with the efficient application of next-generation sequencing technologies to genome sequencing, reference genomes of representative and important species in a broad spectrum of organisms are acquired, being sequenced, and re-sequenced. It becomes important that tools for assembling re-sequenced genomes from high-throughput data are readily available and specifically tuned to particular data types, such as those from ligase-based or polymerase-based protocols [1]. Most currently available assembly tools have been designed for *de-novo* genome assembly, such as Velvet [2]. Recently, several new tools are under development for re-sequencing projects. For example, LOCAS is designed for low coverage assembly of eukaryotic genomes [3]. A commercial tool box developed for re-sequencing projects based on the Roche 454 sequencing platform is designed to assemble both *de-novo* and re-sequencing data. Here, we report a homology-guided method as a new *r*e-sequencing *a*ssembly *t*ool named BIGrat and its testing results for improving the output of the commercial tool Newbler. We believe that BIGrat will be widely used and integrated to the pipeline of next-generation sequencing projects.

## Findings

### The test datasets

Data for assembling rice chloroplast (cp), mitochondrial (mt), and nuclear genomes are all from a genome re-sequencing project for a rice cultivar *PA64S* (*Oryza sativa* L.) [4]. Data for bacterial genome assembly are from *Acinetobacter baumannii* MDR-ZJ06 [5].

### Program design

**Figure 1:**
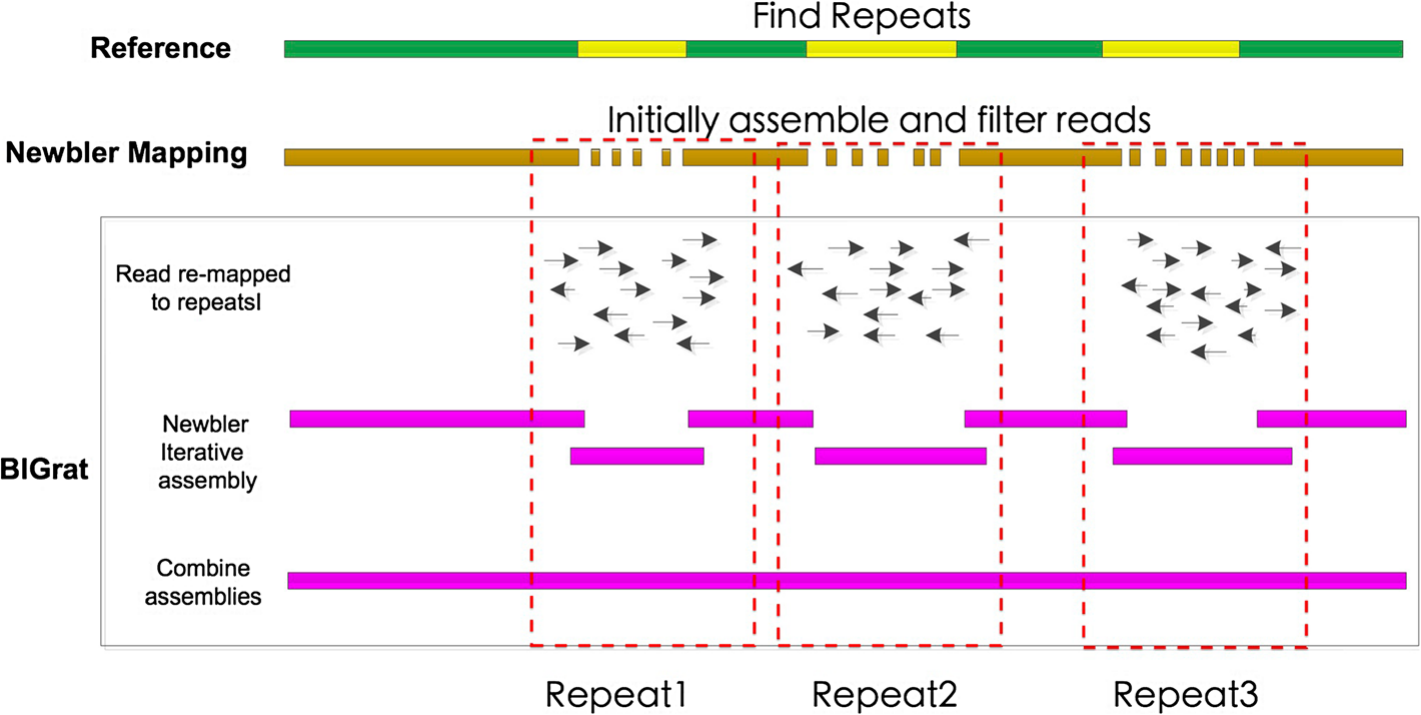
The assembly pipeline of Newber-BIGrat.

BIGrat is based on the mapping result of Newbler and its mapping model. Newbler is not able to assemble repeat sequences in the reference genome correctly and produces many small contigs separated by repeat regions (Additional file 1: Figure S1) but the reads in each repeat region can be assembled separately to completion. Therefore, BIGrat separates the repeat regions with a fixed gap size, and assemble every repeat region iteratively with mapped reads (Figure 1). Such an iterative assembly method has been used in IMAGE [6] and LOCAS [3].

### Program algorithm

First, we use Newbler to mapping the raw data to reference genome and the mapping result will in a file named “454AllContigs.fna”, which stands for the assembled contigs. In order to keep the good and large assembled contigs, in which it means less repeat sequences than rest, we filter the contigs smaller than a gap size (such as 1 kb) but record the those contig coordinates as repeats in the reference genome. In addition, a file named “454PairAlign.txt” also presents in the mapping result and includes all the mapped reads and position in the reference genome. Second, we filter all the reads belong to each repeat in the reference genome and re-assembler each repeat separately to get the new contigs. Normal, the new contigs will better than the filtered one and have a complete repeat region. Last, we combine the initial good assembled contigs and the new contigs in repeats. This can be done with the raw data aligned to the each end of those contigs. We find the overlap in the ends of those contigs and construct the consensus sequences as the last contigs.

## Results and discussion

### Program comparison and assessment

**Figure 2:**
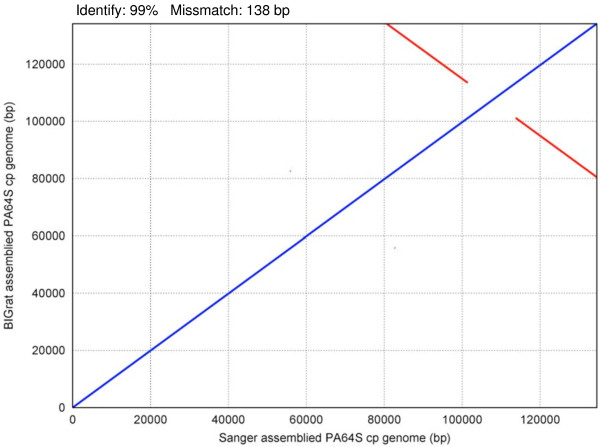
**Dot matrix alignment of PA64S cp genomes between the assembly based on data from the Sanger method and the assembly based on Newbler-BIGrat and Roche 454 data.** The blue and red lines show direct and reverse matches, respectively.

**Figure 3:**
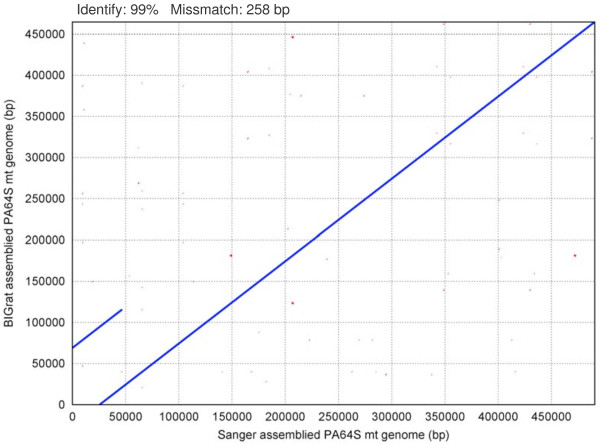
**Dot matrix alignment of PA64S mt genomes between the assembly based on data from the Sanger method and the assembly based on Newbler-BIGrat and Roche 454 data.** The blue and red linesshow direct and reverse matches, respectively.

To evaluate the performance of BIGrat, we used four different genomes against Newbler with its default parameter settings. In addition, we compared assembled results with consensus sequences from BWA-SW/SAMtools [7]. The four genomes are re-sequencing projects carried out at the Beijing Institute of Genomics (BIG) and the assembly results are summarized in Table 1. In the *PA64S* nuclear genome assembly, BIGrat has a better NG50, 19,383 vs. 28,677 bp. BIGrat closed 32.4% of the gaps left by Newbler, with a total length of 8,267,167 bp, and the improvement appears in the contig building (Additional file 2: Figure S2). Moreover, in the rice organellar genome assemblies, BIGrat has also improved the output of Newbler. The chloroplast genome has a typical large repeats [8] and there are also some large repeats in the mitochondrial genome [4]. To look into accuracy and reliability, we compared BIGrat assemblies from rice chloroplast and mitochondrial genomes with the results described in our early publications based on data generated by using the Sanger method [4,9]. The excellent consistency and colinearity between the results produced based on the two methods are rather obvious (Figures 2 and 3). We also tested BIGrat on several bacterial genome projects. For instance, for *Acinetobacter baumannii* MDR-ZJ06, we filled 12% more gaps (32,715 bp) with BIGrat as compared to what Newbler did. Because of the variable repeat contents of eukaryotic genomes, the effectiveness of BIGrat’s sequence assembly is rather different as we showed in the four representative genomes.

**Table 1:** The performance of Newbler and Newbler-BIGrat in assembling different genomes

***Reference***^***1***^	***Assembly method***	***Genome size (bp)***	***Contig length (bp)***	***Contig number***	***Contig NG50***^***2***^	***Contig LG50***^***3***^	***Gap-filling number***^***4***^	***Gap-filling length***^***4***^
Rice PA64S nuclear	BWA-SW	372,317,567	328,243,169	55,092	17,903	4,534	\	\
	Newbler		353,856,308	61,922	19,383	5,351	\	\
	Newbler-BIGrat		362,123,475	41,838	28,677	3,671	20,084	8,267,167
Acinetobacter baumannnii	BWA-SW	3,991,133	3,681,865	133	101,163	14	\	\
	Newbler		3,684,532	119	128,034	12	\	\
	Newbler-BIGrat		3,717,247	104	173,210	9	15	32,715
Rice PA64S mt	BWA-SW	490,673	405,286	5	234,879	1	\	\
	Newbler		273,171	104	1,022	13	\	\
	Newbler-BIGrat		464,774	1	464,774	1	103	191,603
Rice PA64S cp	BWA-SW	134,551	133,024	3	58,368	2	\	\
	Newbler		113,344	56	81,038	1	\	\
	Newbler-BIGrat		134,156	1	134,156	1	55	20,812

Note:

1. The rice reference is a set of pseudomolecules from Rice Genome Annotation Project (version 6.1) ( http://rice.plantbiology.msu.edu) for nuclear, mt and cp genomes. The Acinetobacter baumannii reference genome is the complete sequence from isolate ACICU (accession number: NC_010611). 2. NG50 is the contig length at which 50% of the total genome length is covered. 3. LG50 is the contig number at which 50% of the total genome length is covered. 4. Comparison between Newbler and Newbler-BIGrat.

### Program parameter

BIGrat separates repeat regions in the reference sequence, iteratively fills the gaps caused by the repeats, and assembles the sequence to completion at the end. The main parameter setting is the gap size that is the sum of reassembled repeat regions. We test this parameter from 30 bp to 10,000 bp in *PA64S* chromosome 1. The result showed that 500 bp is an optimal gap size for BIGrat assembly (Additional file 3: Figure S3). This gap size can also be determined based on the sequencing read length. Since the read lengths of the pyrosequencing platforms are ~500 bp from Roche 454 and ~200 bp from IonTorrent, most of the repeats smaller than 200 bp or 500 bp may be assembled based on sequencing reads alone. As the gap size grows, the BIGrat’s running time also increases linearly. For example, the system running times are 54 min, 102 min, and 126 min when gap sizes change from 30 bp to 500 bp and 10,000 bp, respectively.

### Program performance

**Figure 4:**
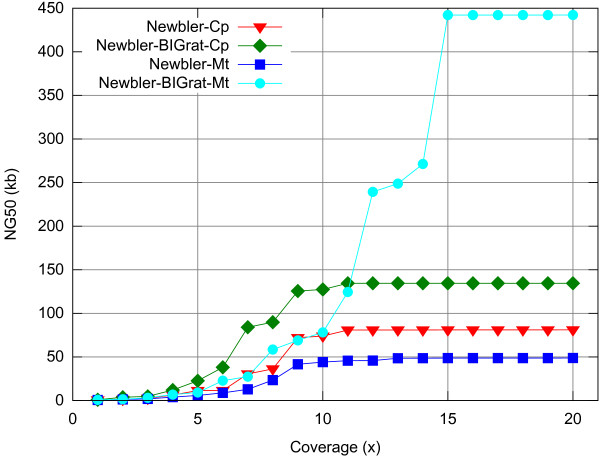
NG50 comparison with different data coverage in the assemblies of rice PA64S chloroplast and mitochondrial genomes based on Newbler and Newbler-BIGrat.

We also implement different data coverage to evaluate BIGrat’s performance by randomly sampling different coverage from 1x to 20x, using the rice chloroplast and mitochondrial genomes as examples (Figure 4). Although the Newbler results showed that increasing data coverage provided little help to improve the assembly when data coverage increased to 10x, our BIGrat assembled the genomes completely as data coverage increased; the chloroplast and mitochondrial genomes were assembled to completion at 10x and 15x coverage, respectively. The results also provide an initial estimation as to what data coverage is needed in genome re-sequencing projects for the two organellar genomes.

## Conclusions

We illustrated an informatics tool BIGrat ( Additional file 4) to improve genome assemblies for pyrosequencing-based re-sequencing projects and showed that BIGrat is an add-on tool to Newbler. BIGrat is easily to be integrated into Newbler for next-generation sequencing assembly and analysis. Because of the limitation to pyrosequencing data and Newbler software, we will update BIGrat software to improve assembly results from all sequencing platforms in next step.

## Availability and requirements

Project name: BIGrat

Project home page: http://sourceforge.net/projects/bigrat/

Operating system(s): Linux Platform

Programming language: Perl

Other requirements: Newbler (version > 2.3)

License: GNU General Public License

Any restrictions to use by non-academics: -

## Competing interests

The authors declare that they have no competing interests.

## Authors' contributions

TZ has implemented the software and written the manuscript. YL, YC, XL and JY have helped design the study and draft the manuscript. All authors read and approved the final manuscript.

## Supplementary Material

Additional file 1: Figure S1 Base depth distribution over the rice chloroplast genome based on Newbler. The contigs are shown as vertical black bars. Click here for file

Additional file 2: Figure S2 Contig comparison between the assemblies of Newbler and Newbler-BIGrat. NG(X) is the contig length at which total genome length is covered X%. Click here for file

Additional file 3: Figure S3 Assembly comparison in the genome of rice PA64S chromosome 1 with different gap-size parameter based on BIGrat’s assembly. The key shows the gap size and time in minute. Click here for file

Additional file 4 **Source code of BIGrat.** See the enclosed README for more information. Click here for file
