# Supplementary figures and images for: BIGrat: a repeat resolver for pyrosequencing-based re-sequencing with Newbler
Source: BMC Res Notes. 2012 Oct 15;5:567. doi: 10.1186/1756-0500-5-567 (PMC3599625; doi:10.1186/1756-0500-5-567)

Base Depth

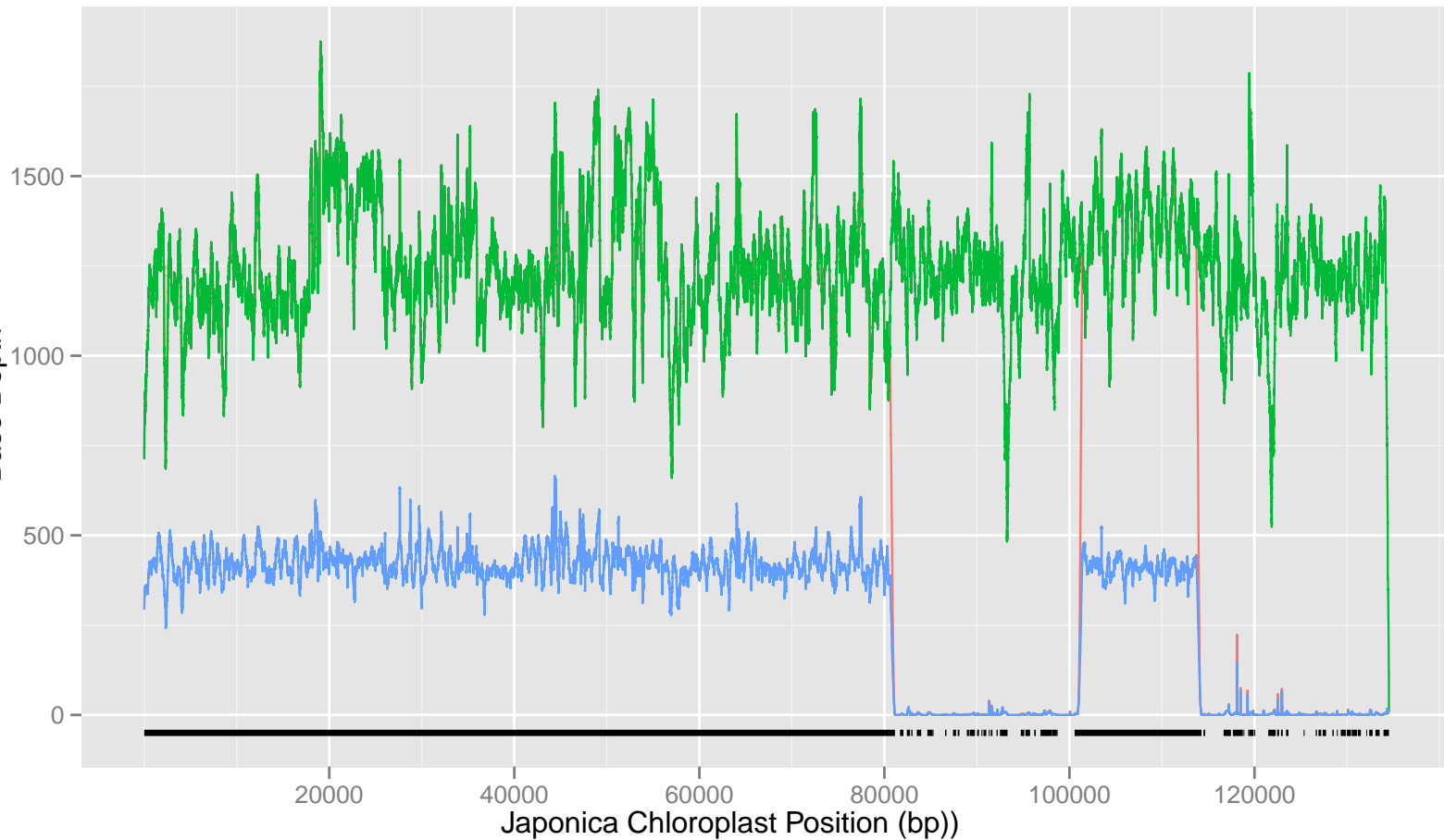

**factor(Depth)**

- Align Depth
- Total Depth
- Unique Depth

Supplement: Additional file 1: Figure S1 — Base depth distribution over the rice chloroplast genome based on Newbler. The contigs are shown as vertical black bars. [file 1756-0500-5-567-S1.pdf]

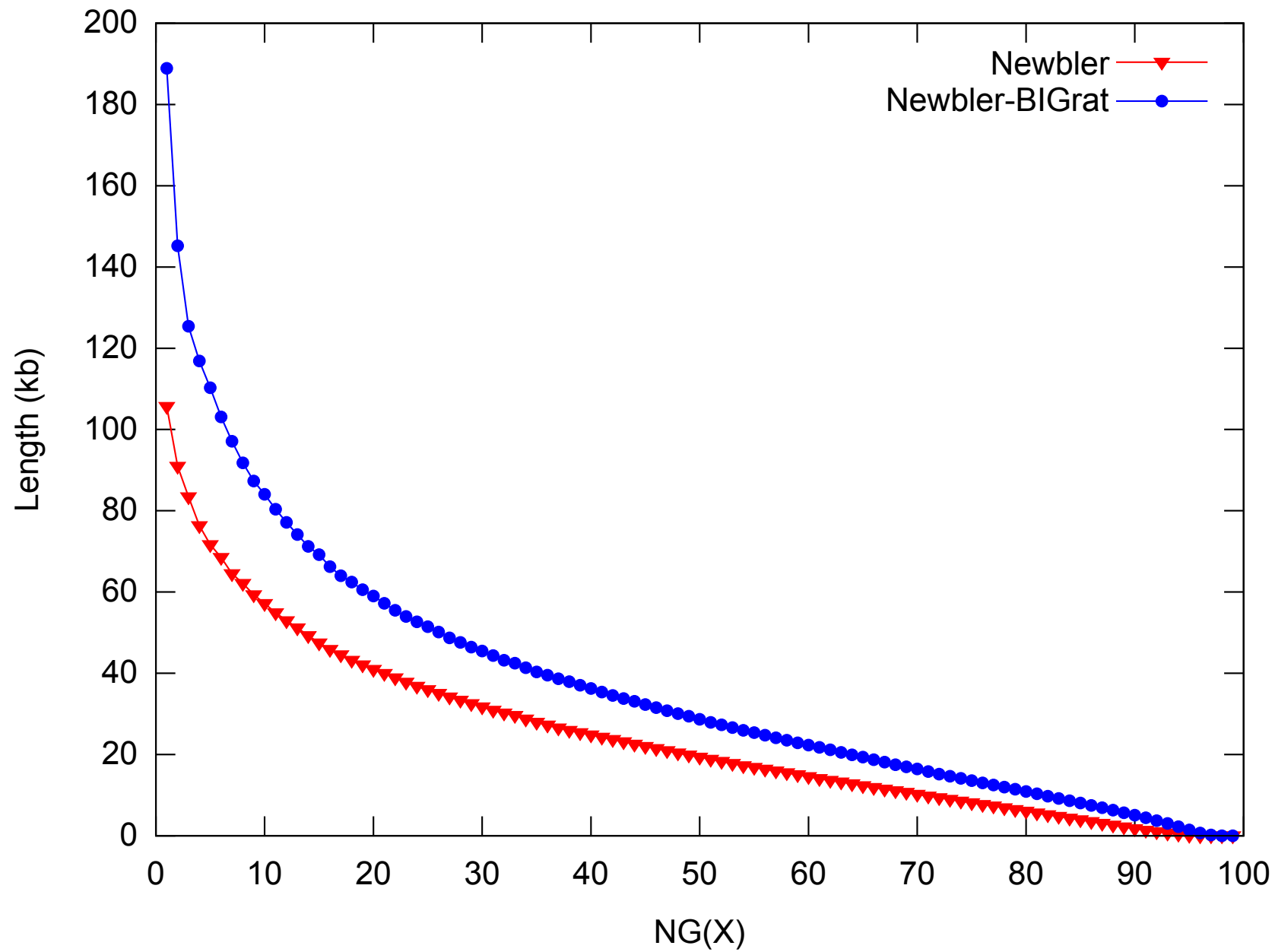

Supplement: Additional file 2: Figure S2 — Contig comparison between the assemblies of Newbler and Newbler-BIGrat. NG(X) is the contig length at which total genome length is covered X%. [file 1756-0500-5-567-S2.pdf]

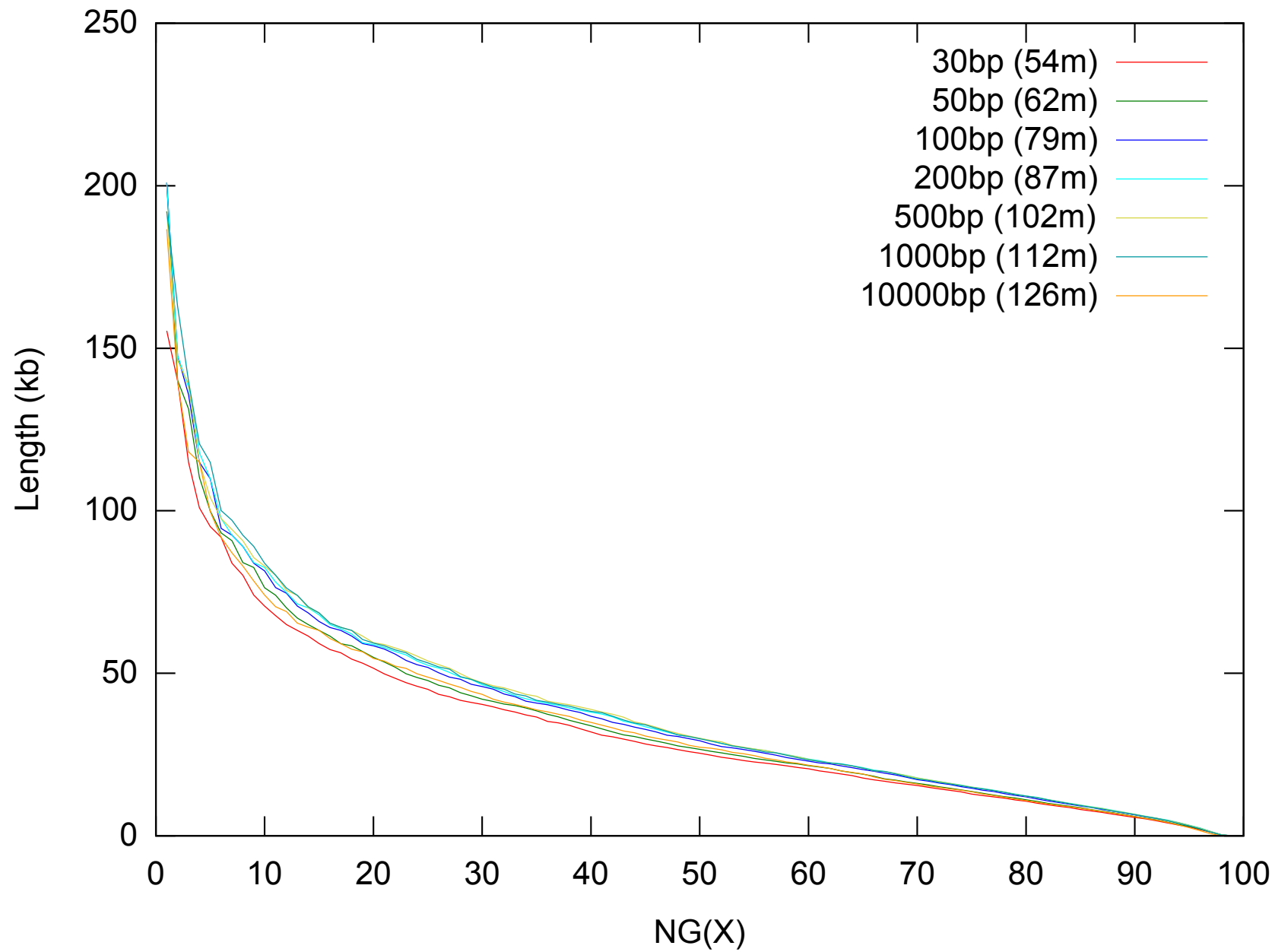

Supplement: Additional file 3: Figure S3 — Assembly comparison in the genome of rice PA64S chromosome 1 with different gap-size parameter based on BIGrat’s assembly. The key shows the gap size and time in minute. [file 1756-0500-5-567-S3.pdf]
